# Clinical Spectrum and Epidemiology of Human Parechovirus Infections in Infants: A Retrospective Study in the Western Part of Sweden

**DOI:** 10.1093/ofid/ofae268

**Published:** 2024-05-14

**Authors:** Karolina Rembeck, Kristina Elfving, Marianela Patzi Churqui, Fredy Saguti, Marie Studahl, Heléne Norder

**Affiliations:** Department of Infectious Diseases, Institute of Biomedicine, Sahlgrenska Academy, University of Gothenburg, Gothenburg, Sweden; Department of Infectious diseases, Sahlgrenska University Hospital, Region Västra Götaland, Gothenburg, Sweden; Department of Pediatrics, Sahlgrenska University Hospital, Region Västra Götaland, Gothenburg, Sweden; Department of Pediatrics, Institute of Clinical Sciences, Sahlgrenska Academy, University of Gothenburg, Gothenburg, Sweden; Department of Infectious Diseases, Institute of Biomedicine, Sahlgrenska Academy, University of Gothenburg, Gothenburg, Sweden; Department of Clinical Microbiology, Sahlgrenska University Hospital, Region Västra Götaland, Gothenburg, Sweden; Department of Infectious Diseases, Institute of Biomedicine, Sahlgrenska Academy, University of Gothenburg, Gothenburg, Sweden; Department of Infectious Diseases, Institute of Biomedicine, Sahlgrenska Academy, University of Gothenburg, Gothenburg, Sweden; Department of Infectious diseases, Sahlgrenska University Hospital, Region Västra Götaland, Gothenburg, Sweden; Department of Infectious Diseases, Institute of Biomedicine, Sahlgrenska Academy, University of Gothenburg, Gothenburg, Sweden; Department of Clinical Microbiology, Sahlgrenska University Hospital, Region Västra Götaland, Gothenburg, Sweden

**Keywords:** human parechovirus, meningoencephalitis, viral sepsis, neonatal meningoencephalitis, neonatal sepsis

## Abstract

**Background:**

Human parechovirus (HPeV) infections can cause sepsis and meningoencephalitis in infants. To improve our knowledge of the consequences of HPeV infections in young children, the incidence, clinical spectrum, and short-term outcome among infants infected with HPeV were investigated retrospectively.

**Methods:**

The presence of HPeV RNA was investigated by polymerase chain reaction in cerebrospinal fluid from 327 children aged 0 to 12 months sampled between 2014 and 2017. Eighty-one were infected with HPeV and included in the study. These infants were divided into 3 groups based on clinical assessment: HPeV was the presumed cause of disease (n = 35); HPeV could have contributed to or been considered the cause of disease (n = 24); and HPeV was not considered the cause of disease (n = 22).

**Results:**

Infection with HPeV type 3 was common in all groups (n = 54), and most children were younger than 3 months (n = 63). The children in the first group (HPeV as presumed cause) had meningoencephalitis (n = 20), viral sepsis (n = 9), or non-severe viral infection (n = 6). The youngest were more prone to develop meningoencephalitis, while the slightly older children had symptoms of viral sepsis or nonsevere viral infection (*P* < .05). Eleven had symptom onset within 2 days after birth. Two infants diagnosed with sudden infant death syndrome were HPeV infected when tested postmortem.

**Conclusions:**

HPeV infections were identified in 25% of children with suspected central nervous system infection. The clinical presentation of those infected with HPeV varied with age. HPeV infections may be associated with sudden infant death syndrome, although this is not well studied. The results suggest that HPeV infections may be underdiagnosed in young infants.

## Teaser

Human parechovirus infection was a common cause of meningoencephalitis and viral sepsis. The clinical presentation varied with age, with the youngest children more often developing meningoencephalitis while the slightly older children had symptoms of viral sepsis or a nonsevere viral infection.

Human parechovirus (HPeV) is increasingly becoming a recognized cause of sepsis and meningoencephalitis (ME) in young infants [1–3]. Parechoviruses are classified into 6 species (parechovirus A–F). Those infecting humans are classified as parechovirus A, which is formed by HPeV types 1 to 19, where type 3 is most commonly associated with sepsis and ME in children younger than 3 months [2, 3]. The clinical spectrum of HPeV infections includes pneumonia, myocarditis, gastroenteritis, as well as asymptomatic infection [4–7]. In neonates, severe ME caused by HPeV may lead to intracranial bleeding, white matter changes, and/or leukoencephalopathy with postinfectious sequelae such as cerebral palsy, epilepsy, and gross motor and cognitive dysfunction [1, 8–11]. HPeV infection has also been associated with sudden infant death syndrome (SIDS) [12, 13].

HPeV transmission is mainly through the fecal-oral and/or respiratory route and most probable through the placenta from mother to fetus [8, 14, 15]. The virus has a biennial peak endemic prevalence, with most cases in temperate climate reported in summer and early fall [10, 16, 17]. To improve our knowledge of HPeV infections and epidemiology from a clinical context, we conducted a retrospective study to investigate the incidence, clinical spectrum, and short-term outcome among infants in our region (Västra Götaland, Sweden) who were HPeV infected during 4 years.

## METHODS

### Inclusion and Study Design

**Figure 1.**
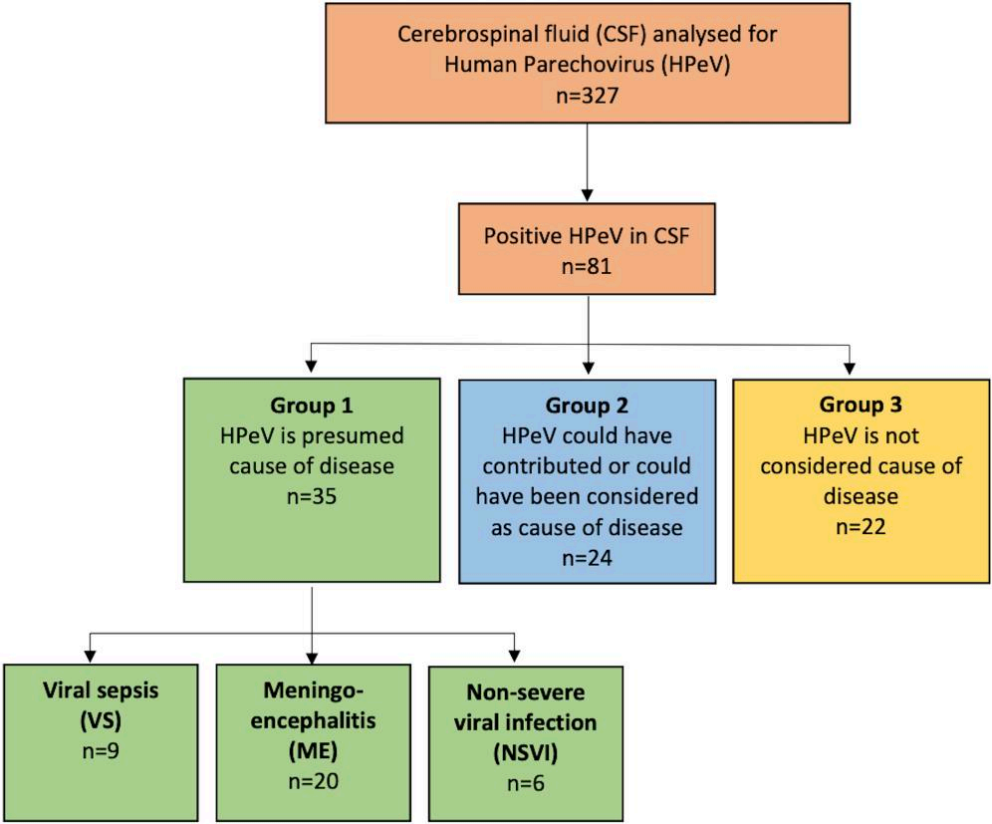
Flowchart and study design. Eighty-one CSF samples with detectable HPeV. Each CSF sample belonged to 1 patient. After review of medical charts, patients were divided into groups 1 (n = 35), 2 (n = 24), and 3 (n = 22). The patients with HPeV as the presumed cause of disease (group 1, n = 35) were further divided by clinical presentation, having VS (n = 9), ME (n = 20), or NSVI (n = 6). CSF, cerebrospinal fluid; HPeV, human parechovirus; ME, meningoencephalitis; NSVI, nonsevere viral infection; VS, viral sepsis.

This retrospective study was based on detection of HPeV in cerebrospinal fluid (CSF) by polymerase chain reaction (PCR). The CSF samples included in the study were drawn from children 0 to 12 months of age with suspected central nervous system (CNS) infection and sent to the Clinical Microbiology Laboratory at Sahlgrenska University Hospital between 1 January 2014 and 20 April 2017. CSF samples were tested for neurotropical viruses as specified by physicians and analyzed by PCR including herpes simplex virus type 1 and 2, varicella zoster virus, cytomegalovirus, Epstein-Barr virus, enterovirus, and/or human herpesvirus 6. Only children admitted to hospitals in Västra Götaland County in Sweden were included, and their stored CSF samples were retrospectively analyzed for HPeV by PCR (see PCR Amplification and Sequencing of Parechovirus Strains). In this study, HPeV was not tested in the clinical routine, since no diagnostic method detecting HPeV in CSF samples was available in the laboratory at that time. The multiplex PCR FilmArray ME panel was implemented in late April 2017, and none of the CSF samples included were tested with the FilmArray ME panel [18]. Only patients with detectable HPeV RNA by PCR in CSF are described in this study (Figure 1). After review of medical journals, a judgment was performed by 3 of the authors (a specialist in infectious diseases, a senior consultant of infectious diseases, and a senior consultant in pediatrics) dividing the patients into 3 groups. Approval from all 3 authors was required for dividing patients into group 1. In group 1, HPeV was the presumed cause of disease; in group 2, HPeV could have contributed to or been considered the cause of disease; and in group 3, the positive HPeV finding was not considered the cause of disease since other diagnoses were more probable. Medical history and radiology, laboratory, and electroencephalography (EEG) results were recorded. The children with presumed HPeV (group 1) were divided into having ME, viral sepsis (VS), or a nonsevere viral infection (NSVI; Figure 1).

### Clinical Definitions

Infants with ME met at least 1 of the following criteria:

- Clinical evidence of meningitis and/or encephalitis, including symptoms of seizure, extensive irritability, or pathologic neurology (altered consciousness and/or focal neurologic signs)
- Reaction Level Scale score ≥3 [19]
- Pathologic EEG
- CSF pleocytosis
- Magnetic resonance imaging, computed tomography, or ultrasound of the brain with pathology in accordance with findings of viral encephalitis.

VS was defined as a combination of irritability and hemodynamic and/or respiratory abnormality and met at least 1 of the following criteria:

- Capillary refill time ≥3 seconds or peripherally cold and marmorated
- Dehydrated or decreased muscle tonus and in need of intravenous fluid
- Hypoxic and in need of oxygen

Less severe infection, with or without fever, was defined as an NSVI. The clinical syndromes are based on previous reports and definitions, and where international consensus is lacking, the definitions are based on clinical experience and local practice [3, 20, 21]. Fever was defined as a temperature ≥38 °C. CSF pleocytosis was defined as CSF with ≥15 × 10^6^ leukocytes/L throughout the first 4 weeks of life, and beyond 1 month of age, >5 × 10^6^ leukocytes/L was considered pathologic [22–26]. Needle bleeding in CSF was handled by reducing 1 leukocyte per 1000 erythrocytes. Tachycardia was defined as a heart rate >165 beats per minute in children aged 0 to 3 months and >155 beats per minute in children aged 4 to 11 months, as defined by the Pediatric Early Warning Score. Premature at birth was, in line with international guidelines, defined as infants born <37 weeks of gestation [27]. Rash included petechiae, exanthema, and a nonspecific rash. Very early onset of symptoms defines a group of neonates with onset within 2 days after birth. Symptom onset within 2 days after birth could include in utero transmission or infection acquired at or during birth. There is no clear consensus on how to define congenital or perinatal acquisition of HPeV, so we settled on defining “very early onset of symptoms” to encompass both.

Nasopharyngeal swab was taken in some patients; these samples were analyzed for the presence of respiratory pathogens by a multiplex PCR panel that targets influenza A and B virus, respiratory syncytial virus, rhinovirus, enterovirus, coronavirus of 4 types (NL63, OC43, 229E, and HKU1), metapneumovirus, adenovirus, parainfluenza virus, and bocavirus, as well as the bacteria *Chlamydophila pneumoniae*, *Mycoplasma pneumoniae*, and *Bordetella pertussis* [28, 29].

### PCR Amplification and Sequencing of Parechovirus Strains

To identify the different parechovirus species, including HPeV types, nested PCRs were performed targeting the VP1 region and the 5′ untranslated region (5′UTR). Total nucleic acid was extracted from 100 µL of CSF and/or serum samples with the DNeasy Blood and Tissue Kit (Qiagen) according to the manufacturer's instructions. The viral nucleic acids were eluted with RNase-free water to a final volume of 200 µL. Before the 5′UTR was amplified, cDNA was synthetized with 5 µL of RNA and 15 µL of the reaction mix containing random hexamer primers and the superscript III RT enzyme (Invitrogen).

The PCR amplifications were performed with the Arktik thermal cycler instrument (Thermo Fisher Scientific), with all the samples tested in duplicate. Each PCR reaction contained 5 µL of the cDNA or the first-round PCR and a 45-µL reaction mixture composed of 31.4 µL of RNase-free water, 10× PCR Buffer II, 2.7mM MgCl_2_ (Applied Biosystems), 0.2mM dNTP (Roche Applied Science), 0.3µM of each primer, and 1 U of Taq polymerase (Roche Applied Science). Amplification was performed for the VP1 region with the primers described by Nix et al [30]. The PCR procedures were initiated at 94 °C for 3 minutes, followed by 40 cycles of amplification (denaturation at 94 °C for 20 seconds, annealing at 54 °C for 30 seconds, and extension at 72 °C for 60 seconds), a final extension at 72 °C for 5 minutes, and holding at 4 °C. For the 5′UTR, primers HPeV253S1 (GGGTGGCAGATGGCGTGCCATAA) and HPeV283R1 (CCTRCGGGTACCTTCTGGGCATCC) were used for the first amplification. For the subsequent nested PCR, primers HPeV313S2 (YCACACAGCCATCCTCTAGTAAG) and HPeV556R2 (GTGGGCCTTACAACTAGTGTTTG) were used, with 5 µL of the first amplification as a template.

After amplification, all the amplicons identified as positive were extracted/purified by the Qiaquick purification kit (Qiagen) according to the manufacturer's instruction. The purified fragments were sequenced with sense and reverse primers in the PCRs and the BigDye Terminator 3.1 (Applied Biosystems) cycle sequencing kit by Sanger sequencing via the Mix2seq service (Eurofins Genomics). All sequences were analyzed with the SeqMan Ultra program in the DNAstar Lasergene package version 17.0 (DNASTAR). The strains amplified and sequenced in the VP1 region were typed by phylogenetic analysis with all reference parechovirus strains. The 5′UTR sequences of these strains were thereafter used for typing the strains in this study that could not be amplified in the VP1 region by phylogenetic analysis of the 5′UTR.

### Statistics

Continuous variables were presented as median and range. Categorical variables were compared by a χ^2^ test or Fisher exact test. Continuous variables were analyzed with 1-way analysis of variance and/or Kruskal-Wallis via Prism (GraphPad Prism version 10.2.2). *P* < .05 was considered statistically significant.

## RESULTS

Out of 327 CSF samples analyzed from 327 patients with a median age of 22 days, 81 (25%) had detectable HPeV RNA. Only 11 strains could be sequenced in the VP1 region and 5′UTR. The sequence homologies of the 5′UTR between these typed strains and 70 strains that could not be amplified in VP1 formed clades based on the virus type. The sequenced strains that could be found in a clade with a known type were considered typable. In 35 (43%) out of the 81 children, HPeV was the presumed cause of disease (group 1); in 24 children (30%), HPeV infection could have contributed to or been considered the cause of disease (group 2); and in 22 children (27%), HPeV was not considered the cause of disease (Figure 1). Baseline characteristics are described in Table 1. In general, most children were younger than 3 months (n = 63, 78%) and infected by HPeV type 3 (n = 54, 67%). Tachycardia was more common in group 1 (*P* = .002), and group 3 had the highest C-reactive protein levels (*P* = .01). There was a peak of HPeV infections in 2014, and most cases throughout the years of 2014 to 2017 were registered in January and February (n = 12 each), followed by June (n = 9) and April (n = 8). The lowest number of cases was noted in August (n = 1) and December (n = 3; more detailed information in Supplementary Figure 1).

**Table 1.** Baseline Characteristics in Patients in Groups 1, 2, and 3

	Patients,^a^ No. (%) or Median (Range)				
	Total (N = 81)	Group 1 (n = 35)	Group 2 (n = 24)	Group 3 (n = 22)	*P* Value
Gender					.1
** **Female	44	20 (57)	16 (67)	8 (36)	
** **Male	37	15 (43)	8 (33)	14 (64)	
Age, mo					
** **0–2	63 (78)	28 (80)	18 (75)	17 (77)	.9
** **3–5	6 (7)	4 (11)	1 (4)	1 (5)	.5
** **6–12	12 (15)	3 (9)	5 (21)	4 (18)	.4
HPeV type					
** **1	8 (10)	4 (11)	1 (4)	3 (14)	.5
** **2	1 (1)	1 (3)	…	…	.1
** **3	54 (67)	24 (69)	15 (63)	15 (68)	.9
** **4	…	…	…	…	NA
** **5	1 (1)	…	1 (4)	…	.6
** **6	4 (5)	3 (9)	1 (4)	…	.5
** **Not typed	13 (16)	3 (9)	6 (25)	4 (18)	NA
CRP, mg/L ^b^					
** **>5	42 (52)	23 (66)	6 (25)	13 (59)	**.006**
** **Highest value	21 (<5–460)	11 (<5–215)	<5 (<5–250)	41 (<5–460)	**.01**
CSF					
** **Pleocytosis ^c^	24 (30)	12 (34)	7 (29)	5 (23)	.9
** **WBC count, x 10^6^/L ^c^	3 (<3–1111)	3.5 (<3–1111)	<3 (<3–317)	3 (<3–73)	.5
** **Protein, g/L ^d^	273 (0.2–2750)	246 (0.2–2000)	324 (0.2–1250)	312 (116–2750)	.3
Fever ^e^	38 (47)	18 (51)	7 (29)	13 (59)	.1
Seizures	31 (38)	15 (43)	12 (50)	4 (18)	.06
Irritable	20 (25)	15 (43)	3 (13)	2 (9)	**.01**
Tachycardia per PEWS ^f^	28 (35)	19 (54)	3 (13)	6 (27)	**.002**
Heart rate ^f^	150 (40–236)	175 (80–236)	140 (40–198)	149 (110–220)	**.02**

Bold *P* value indicates significance.

Abbreviations: CRP, C-reactive protein; CSF, cerebrospinal fluid; HPeV, human parechovirus; NA, not applicable; PEWS, Pediatric Early Warning Score; WBC, white blood cell.

^a^Group 1, patients with HPeV as the presumed cause of disease; group 2, HPeV could have contributed to or been considered the cause of disease; group 3, the positive HPeV finding was not considered the cause of disease.

^b^6 missing values.

^c^2 missing values.

^d^15 missing values.

^e^6 missing values.

^f^12 missing values.

### Group 1: Children With Presumed HPeV-Caused Disease

Of the 35 children in group 1, 20 (57%) had ME, 9 (26%) had VS, and 6 (17%) had an NSVI. Baseline characteristics, symptoms, and laboratory findings in samples from patients in group 1 are described in Table 2. No other neurotropical virus was detected by PCR in CSF, and blood, urine, and CSF bacterial cultures were negative.

**Table 2.** Baseline Characteristics, Symptoms, and Laboratory Data for Patients in Group 1 (HPeV as Presumed Cause of Disease) Divided by Clinical Syndrome

	Group 1 Patients, No. (%) or Median (Range)				
	Total (n = 35)	VS (n = 9)	ME (n = 20)	NSVI (n = 6)	*P* Value
Gender					.9
Female	20 (57)	5 (56)	12 (60)	3 (50)	
Male	15 (43)	4 (44)	8 (40)	3 (50)	
Age, mo					
0–2	28 (80)	7 (78)	17 (85)	4 (67)	.6
3–5	4 (11)	2 (22)	1 (5)	1 (17)	.3
6–12	3 (9)	…	2 (10)	1 (17)	.6
Congenital	11(31)	…	11 (55)	…	**.002**
Premature	10 (29)	4 (44)	5 (25)	1 (17)	.6
CRP, mg/L					
>5	23 (66)	6 (67)	13 (65)	4 (67)	>.99
Highest value	11 (<5–246)	11 (<5–170)	11 (<5–246)	12 (<5–100)	.9
CSF					
Pleocytosis	10 (29)	…	10 (50)	…	NA
WBC count, × 10^6^/L	…	…	12 (<3–1111)	…	NA
Tachycardia ^a^	19 (54)	7 (78)	8 (40)	4 (67)	.1
Heart rate ^a^	175 (80–236)	186 (140–236)	170 (120–202)	175 (80–186)	.1
Fever	18 (51)	7 (78)	7 (35)	4 (67)	.09
Rash	8 (23)	3 (33)	2 (10)	3 (50)	.06
Respiratory distress (apnea included)	16 (46)	3 (33)	12 (60)	1	.1
Irritability	11 (31)	4 (44)	5 (20)	2	.6
Seizure	15 (43)	0	15 (75)	0	NA
Pathologic EEG	7 (20)	0	7 (35)	0	NA
Antibiotics ^b^	29 (83)	8 (89)	18 (90)	3 (50)	.2

Abbreviations: CRP, C-reactive protein; CSF, cerebrospinal fluid; EEG, electroencephalography; HPeV, human parechovirus; ME, meningoencephalitis; NA, not applicable; NSVI, nonsevere viral infection; VS, viral sepsis; WBC, white blood cell.

^a^5 missing values.

^b^1 missing value.

Three children had heart disease, 1 had hip dysplasia, and 1 had multiple-organ malformation. One child had cancer; no other chronic diseases were noted. Twenty-four (69%) children in this group were infected by HPeV type 3 (all ages included). There were 28 children (80%) in this group younger than 3 months, and 19 of them were infected with HPeV type 3.

**Figure 2.**
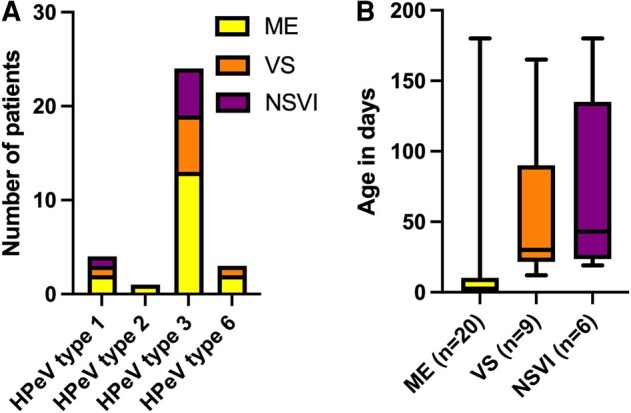
(*A*) HPeV type distribution and (*B*) age at symptom onset (*P* = .0005) in relation to clinical spectrum: ME, VS, or NSVI. *A*, Three were nontypable: 2 in the ME group and 1 in the VS group. *B*, Data are presented as median (line), IQR (box), and range (error bars). HPeV, human parechovirus; ME, meningoencephalitis; NSVI, nonsevere viral infection; VS, viral sepsis.

HPeV type distribution in relation to clinical spectrum is illustrated in Figure 2*A*. Children with ME were younger than children with VS and NSVI, with a median age of 2 days (range, 0–180) at symptom onset as compared with 30 days in the VS group (range, 12–165) and 43 days in the NSVI group (range, 19–180; *P* = .0005; Figure 2*B*). All neonates with very early onset of symptoms had ME (n = 11, *P* = .002). Twenty-nine (83%) received immediate empiric intravenous antibiotics, and 13 were administered empiric intravenous acyclovir (37%).

#### Children With ME

Sixteen (80%) of the 20 children with ME were younger than 1 month, and 11 of those had very early onset of symptoms. In general, the children had a relatively acute onset of disease; only 1 presented with a week of prior symptoms of a cold. Symptoms and laboratory data are presented in Table 2. Ten infants (50%) had CSF pleocytosis: they had a median 25 × 10^6^ leukocytes/L (range, 15–1111), and all but 2 had a predominance of mononuclear leukocytes. Out of these 10 infants, 5 (50%) had CSF needle bleeding where the CSF erythrocyte correction factor was used. Of the 15 children with seizures, 7 (46%) had pathologic EEG results. Eight had repeated seizures, and 2 received anticonvulsive epileptic medication after discharge. Ultrasound or magnetic resonance imaging of the brain was performed in 16 children, and findings were pathologic for 10 of them in terms of intracranial bleeding (n = 5), secondary hydrocephalus (n = 1), ischemic changes (n = 3), and intracranial bleeding and cortical necrosis (n = 1). For 4 patients, follow-up in regard to suspected neurologic sequelae was registered, but only 2 follow-ups were found in the current chart system, where 1 patient had normal findings and 1 had suspected autism.

#### Children With ME and Very Early Onset of Symptoms

**Figure 3.**
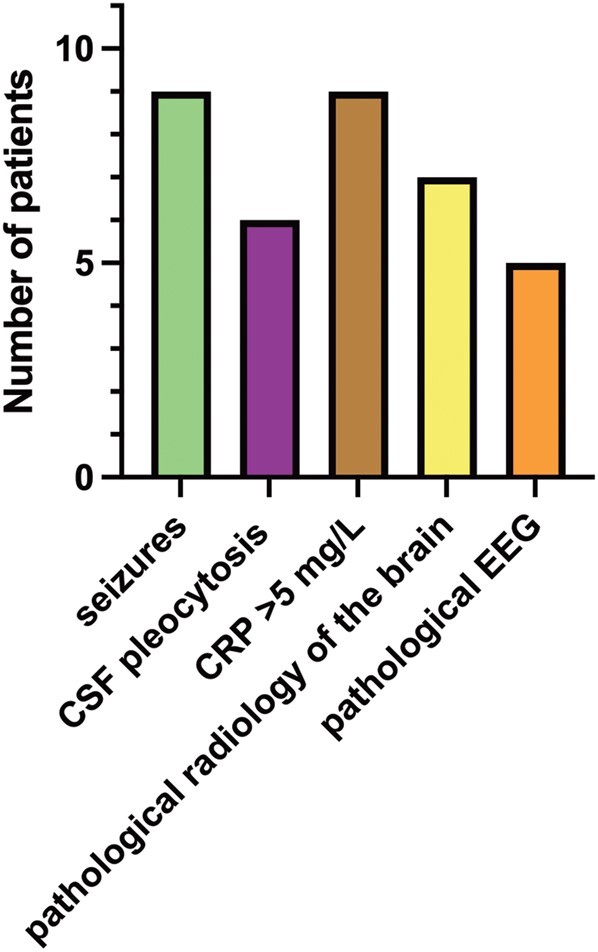
Clinical description of patients with very early onset of symptoms (n = 11). CRP level >5 mg/L is taken during course of care. CRP, C-reactive protein; CSF, cerebrospinal fluid; EEG, electroencephalography.

Eleven infants in this group (group 1 with ME) had onset of symptoms within 2 days after birth. They were mainly infected by HPeV type 3 (n = 7, 63%); 1 each by types 1, 2, and 6; and for 1, the virus stain was not typable. Two were born premature (week 35 + 1 and week 35 + 4, respectively); 2 had meconium-stained amniotic fluid at birth; and 1 was born by vacuum extraction. No other birth or pregnancy complications were noted. Infection in the surrounding family was uncommonly registered; only 1 mother had an upper respiratory tract infection in late pregnancy. Postnatally, 9 children (81%) were described to have some kind of respiratory distress, which included need of oxygen or CPAP/HFNO, tachypnea, or incidents of apnea or cyanosis. A detailed description of this subgroup of patients with very early onset of symptoms is illustrated in Figure 3.

#### Children With VS

Nine children had VS. Eight patients (89%) experienced an acute onset of symptoms, and 1 had 4 days of symptomatic disease prior to hospital admission. In addition to the symptoms described in Table 2, the children were tired and/or lethargic (5/55%), and 2 (22%) had feeding difficulties. Five (56%) received intravenous fluid, and 2 (20%) needed supportive oxygen and/or continuous positive airway pressure/high-flow nasal oxygen. Only 1 was planned for follow-up after discharge secondary to this infection, but information regarding follow-up was not found in the current chart system.

#### Children With NSVI

Six children had an NSVI caused by HPeV. Two (33%) had a cough for 7 days before clinical deterioration; the others had a rather prompt onset of symptoms. Symptoms and laboratory data are presented in Table 2. None of the children were regarded by the attending pediatricians to be in need of follow-up for suspected neurologic sequelae.

### Group 2: Children Where HPeV Could Have Contributed to or Been Considered the Cause of Disease

In 24 patients, HPeV was considered to have contributed to or been considered the cause of disease. Baseline characteristics are described in Table 1. Four had organ malformation at birth. Median age at symptom onset was 43 days (range, 0–290), and 4 had very early onset of symptoms. On symptom onset, 7 children were still in neonatal care or the maternity hospital, and in the majority of the others, there was a relative acute onset of symptoms, with a median time <24 hours from onset of symptoms prior to hospital admission (range, <1 to 14 days). In addition to the symptoms presented in Table 1, 4 (17%) had symptoms of cold or pneumonia, and 3 (13%) had episodes of apnea. The 7 patients with CSF pleocytosis had a median 50 × 10^6^ leukocytes/L (range, 11–317), all with a predominance of mononuclear leukocytes, where 2 lumbar punctures were performed postmortem. Fourteen (58%) received empiric antibiotic treatment, and 5 (21%) were administered empiric acyclovir (4 with combination treatment). Blood and CSF cultures were negative in the samples taken, although 1 had a positive urine culture with growth of *Escherichia coli*; this child’s CSF was also enterovirus positive, and the finding of *E coli* in urine was of uncertain clinical significance. Seven children (29%) were coinfected with other viruses, where the most common coviral infection was with enterovirus (n = 4, all positive for enterovirus in CSF), followed by coronavirus (n = 2, positive in nasopharynx aspirate) and 1 double positive for parainfluenza and coronavirus in nasopharynx aspirate. Notably, 2 children aged 9 and 1.5 months with positive HPeV in CSF died of SIDS, and 1 child died of respiratory failure a few hours after birth.

### Group 3: Children Where the Positive HPeV Finding Was Not Considered the Cause of Disease

In 22 patients, CSF HPeV RNA was not considered clinically significant since other diagnoses were considered more probable. Eleven children had bacterial infections, where most had bacterial meningitis (n = 4, 17%) or sepsis (n = 4, 17%) caused by *Streptococcus agalactiae* (n = 2), *E coli* (n = 1), *Neisseria meningitidis* (n = 1), or other bacteria not verified (n = 4). Bacterial etiology was verified by bacterial culture. One patient had a febrile urinary tract infection (*E coli* in a urine sample). Some of the other causes of disease were infantile spasm (n = 1) and metabolic disorder (n = 1). Median age at onset of symptoms was 25 days (range, 0–340). Data on previous epidemiology on viral symptoms were available in some cases—for example, the child with meningitis caused by meningococcus had a cold a week prior to onset of bacterial meningitis. Symptoms and laboratory data are presented in Table 2.

## DISCUSSION

This study demonstrates that HPeV is a common cause of ME and VS in young infants. We found that most patients with ME or VS caused by HPeV were younger than 3 months and infected by HPeV type 3, which is in line with previous reports [1–3]. Most children had ME, which was expected, since our study was based on CSF samples sent to the Clinical Microbiology Laboratory for analysis of neurotropical virus. Interestingly, in our study, the clinical presentation of HPeV infection varied with age, with the youngest more often developing ME while the slightly older children had symptoms of VS or NSVI (*P* < .05). Eleven patients had a very early onset of symptoms (ie, within 2 days after birth). Very early onset of symptoms implies either viral in utero transmission or perinatally acquired infection. This is not a novel finding, but 11 patients with suspected vertical transmission are probably the largest group reported to date [8, 31], which may indicate that this infection is underdiagnosed in newborns. In a previously reported case of suspected in utero transmission of an HPeV type 3 infection, a boy born preterm (week 32) had a poor visual, motor, and neuropsychological outcome at 2.8 years. Due to the lack of follow-up in regard to suspected neurologic sequelae in the current chart system, we were unable to look into neurologic sequelae in infants with very early onset of symptoms and the others with presumed HPeV infection.

In line with previous studies, the children infected with HPeV in this study had a relatively rapid onset of symptoms and a deteriorating general condition with or without tachycardia [2, 3, 20]. The absence of pleocytosis in the CSF does not exclude CNS infection [1]: in this study, only half of the patients with ME had abnormally high leukocytes in CSF. Our study identified patients with detectable HPeV in their CSF without, in our judgment, having a CNS infection; rather, they were considered to have VS or NSVI. This clinical judgment was based on a lack of clinical symptoms of meningitis or encephalitis, with no CSF pleocytosis nor any radiologic signs of white matter involvement. The presence of HPeV RNA in CSF could be due to viremia, with viral RNA leaking to CSF without causing any CNS infection. This was previously reported by Harvala et al, who compared HPeV viral load in CSF and blood with clinical presentation and found that children with detectable HPeV RNA in CSF without symptoms of CNS disease had 1000- to 10 000-fold higher viral RNA in plasma vs CSF [32]. In this study, such comparison could not be performed due to a lack of serum samples from most patients.

Patients coinfected with other viruses of clinical relevance were classified into group 2—that is, indeterminant of whether HPeV could have caused the disease or contributed to its course. Four children were coinfected by enterovirus, an infection by clinical features indistinguishable from HPeV infection in young infants [33]. The concomitant finding of enterovirus and HPeV in CSF is not novel; perhaps if quantitative PCR in serum and CSF is performed, the possibility to determine a final diagnosis would increase [34]. There is also the possibility that a double viral infection aggravated the disease.

The detection of HPeV in the CSF of 2 children with SIDS and 1 child who died in respiratory failure a few hours after birth is difficult to interpret. This is still an important finding since there are several reports of a suspected association between SIDS and HPeV infection with a fatal outcome [12, 13]. There is a need to further investigate whether these findings are related, as a single detection of HPeV RNA is not sufficient to explain these deaths.

Patients in group 3 had other diagnoses that seemed not to be caused by an HPeV infection, but they still had detectable HPeV in CSF. Laboratory contamination among samples could be excluded, since there were different HPeV sequences in different patient samples. A viral infection, with or without symptoms, in patients with other severe bacterial or metabolic disorders is the most probable explanation for the viral finding in patients in group 3. A positive finding on qualitative or quantitative HPeV PCR should be considered carefully with an individual clinical assessment before withdrawing antibiotics. The interpretation of viral load in CSF, serum, or whole blood needs to be further investigated, as well as defined in relation to symptoms and disease duration.

Most cases of HPeV were found in 2014; however, in contrast to other studies, a biennial endemic peak was not found and there were cases throughout the year, not only during summer months [10, 35]. HPeV type distribution was in line with previous reports, with HPeV type 3 being most prevalent (n = 54, 67%). The other HPeV types identified by sequencing were HPeV types 1, 6, 2, and 5. HPeV type 1 is less frequent but is a reported cause of meningitis, whereas the remaining HPeV types account for the majority of infections worldwide [12, 36–39].

A weakness of this retrospective study is the lack of a predefined protocol. For example, the absence of symptoms is not always presented in the medical chart, and relying on another physician’s clinical judgment might lead to misclassification. Still, after chart review, the obtained clinical information was sufficient for dividing the children into groups and describing different clinical syndromes. Although there are weaknesses of retrospective studies, clinical assessments of retrospective data are necessary to perform to advance interpretation of viral findings. The classification of having ME or VS is of great importance for future studies since postinfectious sequelae are mainly associated with ME [9, 10].

In conclusion, this study demonstrates that HPeV is a common cause of sepsis and ME. Children who are infected are most often younger than 3 months and infected by HPeV type 3. HPeV infection should be considered a cause of disease in young infants with ME or VS throughout the whole year, not only during the summer months. A quantitative PCR test comparing virus levels in blood and serum is warranted in future studies, as well as a thorough history of maternal infection when an in utero transmission or infection acquired at or during birth is suspected. Preferably, fecal samples from the newborns and their mothers should be analyzed for the presence of viruses. This study also demonstrates the importance of a clinical assessment with regard to a positive viral finding.

## Supplementary Material

ofae268_Supplementary_Data

## Supplementary Data

Supplementary materials are available at *Open Forum Infectious Diseases* online. Consisting of data provided by the authors to benefit the reader, the posted materials are not copyedited and are the sole responsibility of the authors, so questions or comments should be addressed to the corresponding author.

## Notes

***Author contributions.*** K. R., H. N., M. S., and K. E. planned and designed the study. K. R., H. N., K. E., and M. S. identified the patients. K. R., K. E., and M. S. collected information about the patients (medical charts) and diagnosed the patients retrospectively according to study criteria. H. N. was responsible for the laboratory method and analysis. H. N., F. S., and M. P. C. were responsible for PCR analysis and sequencing. K. R., K. E., M. S., and H. N. analyzed the data. K. R. wrote the first draft of the manuscript, and K. E., H. N., M. S., F. S., M. P. C., and K. R. edited the manuscript. All authors wrote and approved the final version of the manuscript.

***Patient consent statement.*** The Regional Ethics Review Board in Gothenburg approved the study (Dnr 775-17). The National Ethics Review Board in Sweden approved an amendment (Dnr 2022-03425-02). The study does not include factors necessitating patient consent.

***Financial support.*** This work was supported by the Wilhelm and Martina Lundgrens Researchfund; the C4I from Sahlgrenska University Hospital; and by grants from the Swedish state under the ALF agreement between the Swedish government and country councils (ALFGBG 74050).
