# Supplementary figures and images for: Clinical Spectrum and Epidemiology of Human Parechovirus Infections in Infants: A Retrospective Study in the Western Part of Sweden
Source: Open Forum Infect Dis. 2024 May 14;11(5):ofae268. doi: 10.1093/ofid/ofae268 (PMC11134459; doi:10.1093/ofid/ofae268)

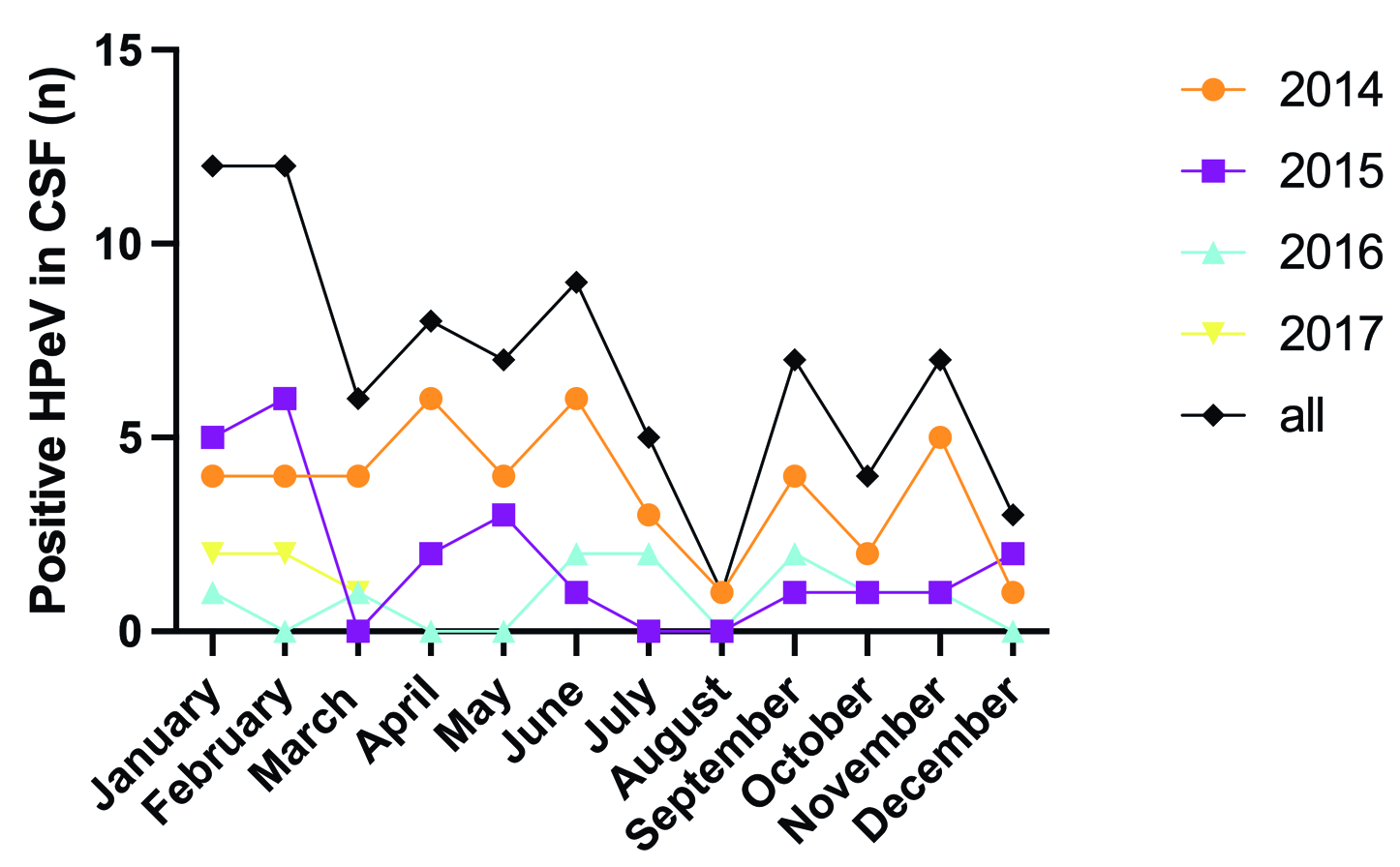

Supplement: ofae268_Supplementary_Data [file ofae268_supplementary_data.zip › Supplementary figure.tiff]
